# Prediction of robotic neurorehabilitation functional ambulatory outcome in patients with neurological disorders

**DOI:** 10.1186/s12984-021-00965-6

**Published:** 2021-12-18

**Authors:** Chao-Yang Kuo, Chia-Wei Liu, Chien-Hung Lai, Jiunn-Horng Kang, Sung-Hui Tseng, Emily Chia-Yu Su

**Affiliations:** 1 Graduate Institute of Biomedical Informatics, College of Medical Science and Technology, Taipei Medical University, 172-1, Sec. 2, Keelung Rd., 10675 Taipei City, Taiwan; 2 Department of Physical Medicine and Rehabilitation, Taipei Medical University Hospital, 252 Wuxing St, Xinyi District, 11031 Taipei City, Taiwan; 3 Department of Physical Medicine and Rehabilitation, School of Medicine, College of Medicine, Taipei Medical University, 250 Wu-Hsing Street, 11031 Taipei City, Taiwan; 4 Clinical Big Data Research Center, Taipei Medical University Hospital, 252 Wuxing St, Xinyi District, 11031 Taipei City, Taiwan; 5 Research Center for Artificial Intelligence in Medicine, Taipei Medical University, 250 Wu-Hsing Street, 11031 Taipei, Taiwan

**Keywords:** Robotic neurorehabilitation, Machine learning, Lokomat, Functional ambulatory outcome, Random forest

## Abstract

**Introduction:**

Conflicting results persist regarding the effectiveness of robotic-assisted gait training (RAGT) for functional gait recovery in post-stroke survivors. We used several machine learning algorithms to construct prediction models for the functional outcomes of robotic neurorehabilitation in adult patients.

**Methods and materials:**

Data of 139 patients who underwent Lokomat training at Taipei Medical University Hospital were retrospectively collected. After screening for data completeness, records of 91 adult patients with acute or chronic neurological disorders were included in this study. Patient characteristics and quantitative data from Lokomat were incorporated as features to construct prediction models to explore early responses and factors associated with patient recovery.

**Results:**

Experimental results using the random forest algorithm achieved the best area under the receiver operating characteristic curve of 0.9813 with data extracted from all sessions. Body weight (BW) support played a key role in influencing the progress of functional ambulation categories. The analysis identified negative correlations of BW support, guidance force, and days required to complete 12 Lokomat sessions with the occurrence of progress, while a positive correlation was observed with regard to speed.

**Conclusions:**

We developed a predictive model for ambulatory outcomes based on patient characteristics and quantitative data on impairment reduction with early-stage robotic neurorehabilitation. RAGT is a customized approach for patients with different conditions to regain walking ability. To obtain a more-precise and clearer predictive model, collecting more RAGT training parameters and analyzing them for each individual disorder is a possible approach to help clinicians achieve a better understanding of the most efficient RAGT parameters for different patients.

*Trial registration:* Retrospectively registered.

**Supplementary Information:**

The online version contains supplementary material available at 10.1186/s12984-021-00965-6.

## Introduction

Neurological disorders are often chronic and debilitating, and place heavy burdens on families and society [1]. Improving mobility is one of the main goals of rehabilitation for patients with neurological disorders [2]. In neurorehabilitation, a high dose and intensity, sufficient practice, individualized goals, motivation, and specialist knowledge are all important factors for achieving better outcomes [3, 4]. Compared to conventional therapy, robotic-assisted gait training (RAGT) is expected to more effectively improve mobility, as it can provide a higher dose and more-intensive treatment than usual rehabilitation [5]. As early as 2009, randomized controlled trials showed that RAGT combined with regular physiotherapy was more effective for improving the functional ambulation capacity and neurological recovery than conventional therapy in patients after a subacute stroke [6].

Comparisons of the efficacy between RAGT and conventional gait training (CGT) have garnered considerable attention in rehabilitation medicine. A review article revealed that RAGT applications demonstrated a better effect than CGT in post-stroke patients [7]. Another review article provided evidence that RAGT improved walking function in patients that sustained a spinal cord injury within the past 6 months [8]. In contrast, other studies demonstrated non-superior results of the effectiveness of RAGT for functional recovery of walking in survivors with different neurological disorders [9, 10]. Variations in the intensity, duration, and amount of training, as well as in the types of treatment, participant characteristics, and measurements across trials may have contributed to different reported effectiveness levels. Nevertheless, specific applications using RAGT devices to obtain optimal effects for patients remain unclear.

Robot-assisted gait trainers operate by either end-effector (non-portable) or exoskeleton (portable) principles. One systematic review showed that operational robots, such as the Gait Trainer, were more cost-effective in achieving independence in walking than wearable robotics, such as Lokomat [11]. However, more than 1000 Lokomat devices have been purchased and are in use worldwide to restore and improve walking function in patients with neurological disorders. The cost and availability of devices like Lokomat often put therapists and patients under considerable pressure as there is still uncertainty regarding their optimal protocol and appropriate timing of use. Further research on the efficient use of Lokomat-assisted therapy is needed.

Periodic outcome assessments and tracking are fundamental approaches for implementing effective medical practices, which are supported by guidelines issued for stroke rehabilitation [12]. A previous study stated that high-intensity step training applied during inpatient rehabilitation resulted in significantly greater walking and balance outcomes [13, 14]. Although RAGT provides highly intensive and repetitive task-specific training, training programs also need to be individualized and monitored for effective neurorehabilitation. However, data obtained from ongoing RAGT can be best used when organized into a predictive model to help clinicians, patients, and their families with decision-making and planning robot-assisted rehabilitation management as early as possible [15].

During Lokomat training, the body weight (BW) support system and guidance force (GF) provided by the robotic arms assist patients to follow a physiological gait pattern. Later, the support and guidance are accordingly reduced as the patient regains selective motor control. Positive relationships between training parameters and muscle activation were also reported. Specifically, during Lokomat training, reducing BW support and GF was shown to increase gluteus muscle and anterior tibialis muscle activation, while increasing the training speed functions to enhance muscle activation in both legs [16, 17]. However, no predictive model based on Lokomat training data has been established to help identify cost-effective approaches for patients using this system to regain walking function.

Therefore, until the phenotypes for an effective intervention are better clarified, Lokomat-based therapy still relies heavily on therapists’ clinical expertise. We hypothesized that early assessment could accurately predict the effectiveness of RAGT for functional gait recovery based on parameters from the first few sessions. The purpose of this study was two-fold. First, we attempted to determine the most beneficial combination of RAGT parameters in adult patients with neurological diseases in different recovery phases. Second, we anticipated the development of prediction models to assess improvements in the Functional Ambulation Category (FAC) for Lokomat-based therapies in this population. FAC, a commonly used outcome measurement in gait-related studies, is a six-point categorical scale that assesses how much support a patient requires when walking. A score of 0 indicates non-walking, while a score of 4 to 5 indicates an increasingly independent walking ability [18].

## Materials and methods

### Dataset

**Fig. 1:**
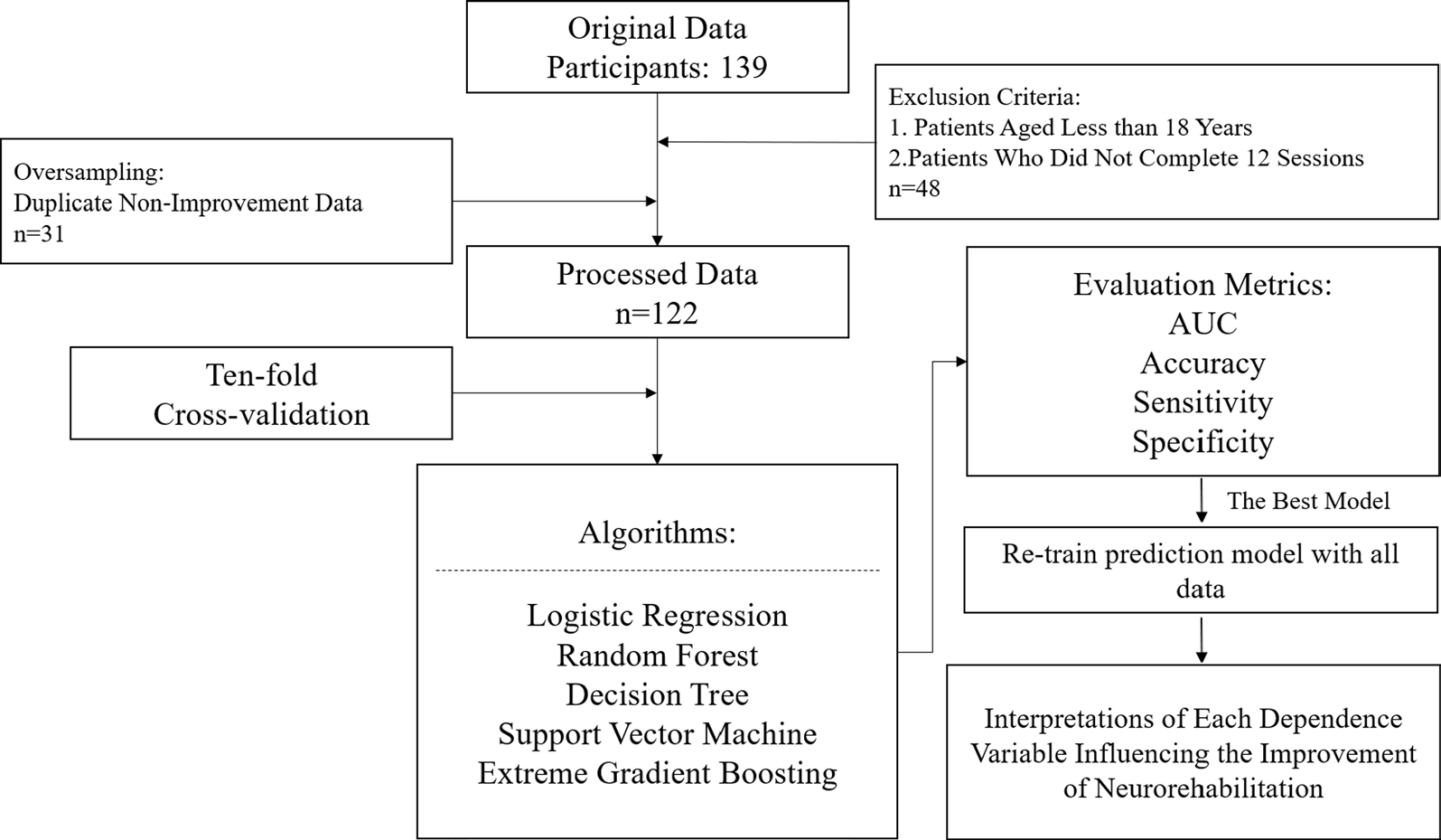
Analytic flowchart to develop models for predicting the effectiveness of robot-assisted gait training (RAGT) for patients with neurological disorders

The analytical flowchart of this study is presented in Fig. 1. Data of 139 patients who underwent Lokomat training at Taipei Medical University Hospital were retrospectively collected. After screening for data completeness, records of 91 adult patients with acute or chronic neurological disorders were included in the study. Clinical information and RAGT parameters of all sessions were incorporated as input variables to predict whether patients would show improvement by comparing the FAC of the 12th session with that of the first session. We incorporated continuous variables (i.e., age, days to complete 12 sessions, BW support, GF, and speed) and categorical variables (i.e., initial FAC, gender, entry point, extremity affected, and diagnosis) as inputs into the prediction models. Among the final dataset, 60 patients (65.9%) showed improvement after completing 12 RAGT sessions, and the remaining 31 patients showed no improvement. Given the importance of walking ability in stroke patients, the RAGT parameters extracted for analysis in this study included BW support, GF, and the speed at which the treadmill was run as they reflect responsive measures of gait ability. These three parameters can individually be adjusted according to a patient’s condition and the therapeutic goals of that patient. At our clinic, therapists develop individualized Lokomat training protocols for each stroke patient. As patients gradually regain strength in their lower extremities, therapists can reduce the weight supported to promote greater muscle activities. When patients regain more temporal muscle activation, the GF can be reduced to promote patients’ active participation in predefined gait patterns. Subsequently, when the performance of a patient adjusts well to the above two parameters, therapists can increase the training walking speed to increase repetitions and challenges. This study was approved by the Institutional Review Board of Taipei Medical University (no. 202,005,039).

### Descriptive statistical analysis

The descriptive statistics were compared between the improvement and non-improvement groups of patients undergoing RAGT treatments (i.e., 60 patients in the improvement group and 31 in the no-improvement group). Means and standard deviations (SDs) were computed for continuous variables, and frequencies and percentages were calculated for categorical variables. Comparisons of baseline characteristics between groups with and without progress were analyzed by Student’s *t*-test for continuous variables and Chi-squared test for categorical variables. Between-group comparisons were considered statistically significant at *p *< 0.05. Statistical analyses were conducted using RStudio vers. 1.2.5001 software (2009–2019 RStudio) and SAS 9.4 (SAS Institute, Cary, NC, USA).

### Imbalanced dataset handling

The original dataset was imbalanced (i.e., 65.9% of participants showed improvement and 34.1% of them did not), and machine learning algorithms tend to predict outcomes of most samples to achieve a better predictive performance. However, one of the most important aims of our study was to identify patients who do not show improvement despite completing many RAGT sessions. Therefore, oversampling of the group with fewer samples, or under-sampling of the group with more samples can be used as an effective technique to handle imbalanced datasets [19]. Therefore, we applied an oversampling technique to double the no-improvement group of patients to generate a final balanced dataset consisting of 60 improved and 62 non-improved participants for development of prediction models in this study.

### Machine learning algorithms

Machine learning is a research field in computer science that focuses on how algorithms learn from data [20]. These algorithms incorporate statistics to detect patterns in order to make predictions about a dataset [21]. The use of machine learning algorithms has become increasingly common for obtaining reliable predictions. Compared to traditional methods, many machine learning techniques and algorithms discover more-sensitive and -specific screening algorithms and relax assumptions and restrictions of traditional regressions [22]. The aim of this study was to build the best prediction model to distinguish patients with and without improvement using RAGT for functional gait recovery. Five machine learning algorithms, including logistic regression (which is widely used in medical studies), decision tree (which generates a tree-like model to support decision making), support vector machine (SVM; which is used to perform nonlinear classification by mapping input features to a higher-dimensional space), random forest (RF), and extreme gradient boosting (XGBoost) to compare performances, were incorporated to predict changes in the FAC and develop prediction models. The logistic regression and SVM are two well-known statistical methods used for binary classification. Furthermore, we used tree-based machine learning algorithms to deal with binary classifications as well. A decision tree is a fundamental tree-based algorithm. RFs and XGBoost are two tree-based algorithms as well, but they fundamentally differ. RFs use bagging to generate each new dataset with replacement from the original dataset. XGBoost is a machine learning algorithm for tree boosting.

Cross-validation was incorporated to provide fair estimations of our prediction model. We used ten-fold cross-validation to evaluate our prediction performance using the balanced dataset (i.e., 60 in the improvement group and 62 in the no-improvement group). Since cross-validation can be a better approach for evaluating predictive performance without overestimation, ten-fold cross-validation was also applied for evaluation of the balanced dataset. The balanced dataset was randomly divided into ten subsets; each time a subset was used as a test set, the remaining nine subsets were applied to develop the prediction model as the training set. We built prediction models and evaluated each model in the R environment.

### Experimental design and prediction models

To investigate the effects of using different numbers of input sessions (i.e., *i* as the number of input sessions), the clinical information and raw parameters from the first *i* RAGT sessions were incorporated as input features in the five machine learning algorithms to predict whether there was an improvement in the 12th session (compared to the 1st session) for the balanced dataset. The number of input sessions with the highest area under the receiver operating characteristic (ROC) curve (AUC) in the test set was selected as the best feature for predicting the success of RAGT. After the optimal number of input sessions was determined, we applied the optimal number of input sessions using ten-fold cross-validation to develop the prediction model for the balanced dataset (i.e., denoted model 1).

Furthermore, instead of predicting whether changes will occur, we investigated whether using a finer granulation to predict the amount of FAC changes as the prediction outcome would improve the performance. The FAC is a six-point categorical scale that assesses the extent to which support is needed by a patient when walking. In addition, changes in the FAC of 0 and 1 accounted for 50.8% and 33.6% (i.e., 62 and 41 patients with FAC changes of 0 and 1, respectively) of the balanced dataset, respectively. Therefore, few samples with FAC changes of ≥ 2 (i.e., 14, 3, 1, and 1 patients with FAC changes of 2, 3, 4, and 5, respectively) were grouped together. We incorporated data from the optimal number of input sessions as features, and FAC changes at three levels (i.e., 0, 1, and ≥ 2) as our outcome target variables to develop a prediction model by ten-fold cross-validation (i.e., denoted model 2).

In addition to an accurate predictive performance, it is highly desirable if clinical insights can be interpreted from experimental results from an analytical study of the data. The RF algorithm estimates the importance of a specific variable by observing how much the error of prediction (i.e., the mean decrease in accuracy, MDA) increases when out-of-bag data for a specific variable are permuted and all other variables remain the same [23].

### Evaluation measures

The accuracy, sensitivity, specificity, and the ROC curve, were used to evaluate the performance of each prediction model. The accuracy, sensitivity, and specificity were defined as follows:

$$Accuracy=\frac{TP+TN}{TP+TN+FP+FN}$$

$$Sensitivity=\frac{TP}{TP+FN}$$

$$Specificity=\frac{TN}{TN+FP}$$

where TP, TN, FP, and FN respectively denote numbers of true positives, true negatives, false positives, and false negatives.

The area under the ROC curve (AUC) is an indicator used to evaluate the performance of the classification prediction models. A previous study suggested that the AUC is a better indicator for comparing and measuring the performance of a classification algorithm [24] because it avoids the assumed subjectivity in threshold selection, while the continuous probability is converted into binary dependent variables and summarizes the performance of each prediction model under all possible thresholds [20]. Therefore, the AUC was used as an evaluation measure to compare different machine learning algorithms and select the classifier with the best performance.

## Results

### Significant differences observed in RAGT parameters in the two study groups

Descriptive statistical analyses of continuous and categorical variables of the 91 patients are shown in Tables 1 and 2, respectively. It was observed that the age of the patient and time to complete one course (i.e., 12 sessions of Lokomat training, in days) did not significantly differ between the improvement and no-improvement groups (Table 2). For Lokomat parameters, all variables except the GF of the 1st session showed significant differences between the two groups. Our analysis also demonstrated that the entry point, extremity affected, and diagnosis showed statistically significant differences in outcomes (Table 1).

**Table 1:** Descriptive statistics of continuous variables for the improvement and no-improvement groups in RAGT

Continuous variables	No- improvement (n = 31)		Improvement (n = 60)		
	Mean	SD	Mean	SD	p -value
Age (years)	61.0000	13.6431	59.0167	15.5514	0.5498
Days of 12 Sessions Accomplished (days)	41.0645	18.7988	36.0833	20.8305	0.2672
GF 1st (%)	90.9677	11.4324	93.2500	9.3349	0.3093
GF 6th (%)	83.5484	13.3662	74.5000	17.3376	0.0128 *
GF 12th (%)	79.3548	17.0657	66.4167	20.6892	0.0036 **
BW support 1st (%)	60.8387	12.1493	50.7333	12.8602	0.0005 ***
BW support 6th (%)	48.2581	13.9115	33.7000	15.4988	0.0000 ***
BW support 12th (%)	42.0645	15.6545	26.2667	15.1343	0.0000 ***
Speed 1st (km/h)	1.4290	0.1216	1.4900	0.1374	0.0401 *
Speed 6th (km/h)	1.5290	0.1346	1.6367	0.0245	0.0024 **
Speed 12th (km/h)	1.5548	0.1748	1.7150	0.2483	0.0006 ***
GF 6th-GF 1st (%)	−7.4194	11.0205	−18.7500	14.8045	0.0003 ***
GF 12th-GF 1st (%)	−11.6129	14.9659	−26.8333	18.4796	0.0002 ***
BW support 6th-BW support 1st (%)	−12.5806	10.6388	−17.0333	10.9312	0.0664
BW support 12th-BW support 1st (%)	−18.7742	12.5531	−24.4667	12.9018	0.0471 *
Speed 6th-Speed 1st (km/hr)	0.1000	0.0931	0.1467	0.1282	0.0760
Speed 12th-Speed 1st (km/hr)	0.1258	0.1437	0.2250	0.1874	0.0116 *

SD, standard deviation; GF, Guidance force; BW, Body weight

*p* < 0.05, ”*”; *p* < 0.01, “**”; *p* < 0.001, “***”

**Table 2:** Descriptive statistics of categorical variables for the improvement and no-improvement groups in RAGT

Categorical variables	No-improvement (n = 31)		Improvement (n = 60)		
	n	%	n	%	p -value
Initial FAC					
0	16	17.5824	19	20.8791	0.0708
1	4	4.3956	12	13.1868	
2	3	3.2967	11	12.0879	
3	2	2.1978	12	13.1868	
4	4	4.3956	6	6.5934	
5	2	2.1978	0	0.0000	
Gender					
Male	22	24.1758	44	48.3516	0.8106
Female	9	9.8901	16	17.5824	
Entry point					
Onset ≤ 3months	4	4.3956	21	23.0769	0.0252 *
> 3 months	27	29.6703	39	42.8571	
Extremity affected					
1	15	16.4835	48	52.7473	0.0020 **
More than 1	16	17.5824	12	13.1868	
Diagnosis					
CVA-hemorrhagic	10	10.9890	22	24.1758	0.0292 *
CVA-ischemic	8	8.7912	22	24.1758	
SCI	12	13.1868	7	7.6923	
TBI	0	0.0000	3	3.2967	
Other neurological Disorders	1	1.0989	6	6.5934	
FAC 6th-FAC 1st					
No-Improvement	31	34.0659	35	38.4615	0.0000***
Improvement	0	0.0000	25	27.4725	

*FAC* Functional ambulation categories, *n* Number

*p* < 0.05, ”*”; *p* < 0.01, “**”; *p* < 0.001, “***”

### Machine learning models accurately predict improvements in the FAC using clinical data and RAGT parameters from all sessions

The predictive performances of different machine learning algorithms with ten-fold cross-validation using different numbers of input sessions to predict the improvement in FACs of the 12th session is shown in Additional file 1: Table S1 in supplementary material. From Additional file 1: Table S1, using all sessions as the input in the RF algorithm to predict the improvement in FACs of the 12th session resulted in achieving the highest AUC of 0.981. In addition, using all sessions as the input in both the logistic regression and XGBoost algorithms resulted in better performances than using other numbers of sessions as the input. Therefore, all sessions were selected as inputs for the machine learning algorithms to further compare prediction performances as shown in Table 3a. The RF achieved high predictive performances of 0.879, 1.000, 0.767, and 0.981 in accuracy, sensitivity, specificity, and AUC, respectively. To investigate whether there were significant differences in the means of the AUC, accuracy, sensitivity, and specificity between the classifiers, an analysis of variance (ANOVA) was incorporated to examine the differences, and our statistical analysis showed that there were significant differences (i.e., *p* < 0.05) in their means among the five classifiers. Detailed statistical results of the ANOVA and Tukey’s honest significant difference (HSD) tests are illustrated in Additional file 1: Tables S2a–S5a and S2b–S5b, respectively.

**Table 3:** Performance for predicting effectiveness of RAGT using (a) Clinical data and all sessions for improvement or not by ten-fold cross-validation, and (b) Using clinical data and all sessions to predict three levels of FAC change by ten-fold cross-validation

Algorithms	Accuracy	Sensitivity	Specificity	AUC
(a) Prediction performance of model 2 using clinical data and all sessions for improvement or not by ten-fold cross-validation				
Random Forest	0.879 ± 0.079	1.000 ± 0.000	0.767 ± 0.161	0.981 ± 0.043
Logistic Regression	0.853 ± 0.101	0.933 ± 0.141	0.767 ± 0.141	0.918 ± 0.061
Support Vector Machine	0.836 ± 0.124	0.855 ± 0.166	0.850 ± 0.123	0.919 ± 0.120
Decision Tree	0.618 ± 0.087	0.626 ± 0.208	0.717 ± 0.284	0.695 ± 0.160
XGBoost	0.854 ± 0.072	0.933 ± 0.141	0.817 ± 0.166	0.937 ± 0.070
(b) Prediction performance of model 3 using clinical data and all sessions to predict three levels of FAC change by ten-fold cross-validation				
Random Forest	0.747 ± 0.074	0.567 ± 0.077	0.846 ± 0.049	0.814 ± 0.093
Logistic Regression	0.535 ± 0.114	0.441 ± 0.147	0.732 ± 0.065	0.675 ± 0.127
Support Vector Machine	0.522 ± 0.132	0.391 ± 0.106	0.720 ± 0.084	0.724 ± 0.131
Decision Tree	0.499 ± 0.065	0.329 ± 0.043	0.667 ± 0.029	0.485 ± 0.038
XGBoost	0.731 ± 0.079	0.580 ± 0.116	0.842 ± 0.051	0.768 ± 0.119

XGBoost, Extreme Gradient Boosting

In addition, we incorporated the same set of features to predict FAC changes of the 12th session compared to all session in model 2. In Table 3b, the means of the AUCs from different machine learning algorithms evaluated by ten-fold cross-validation for three levels were ranked from the highest to the lowest as follows: RF, 0.814; XGBoost, 0.768; SVM, 0.724; logistic regression, 0.675; and decision tree, 0.485. Compared to Table 3a, experimental results demonstrated that using three levels of FAC changes as a finer granulation to estimate whether an outcome variable performed worse than the binary classification of improvement or not were due to the greater number of classes used for the prediction.

### Variable importance ranking and clinical interpretation

**Fig. 2:**
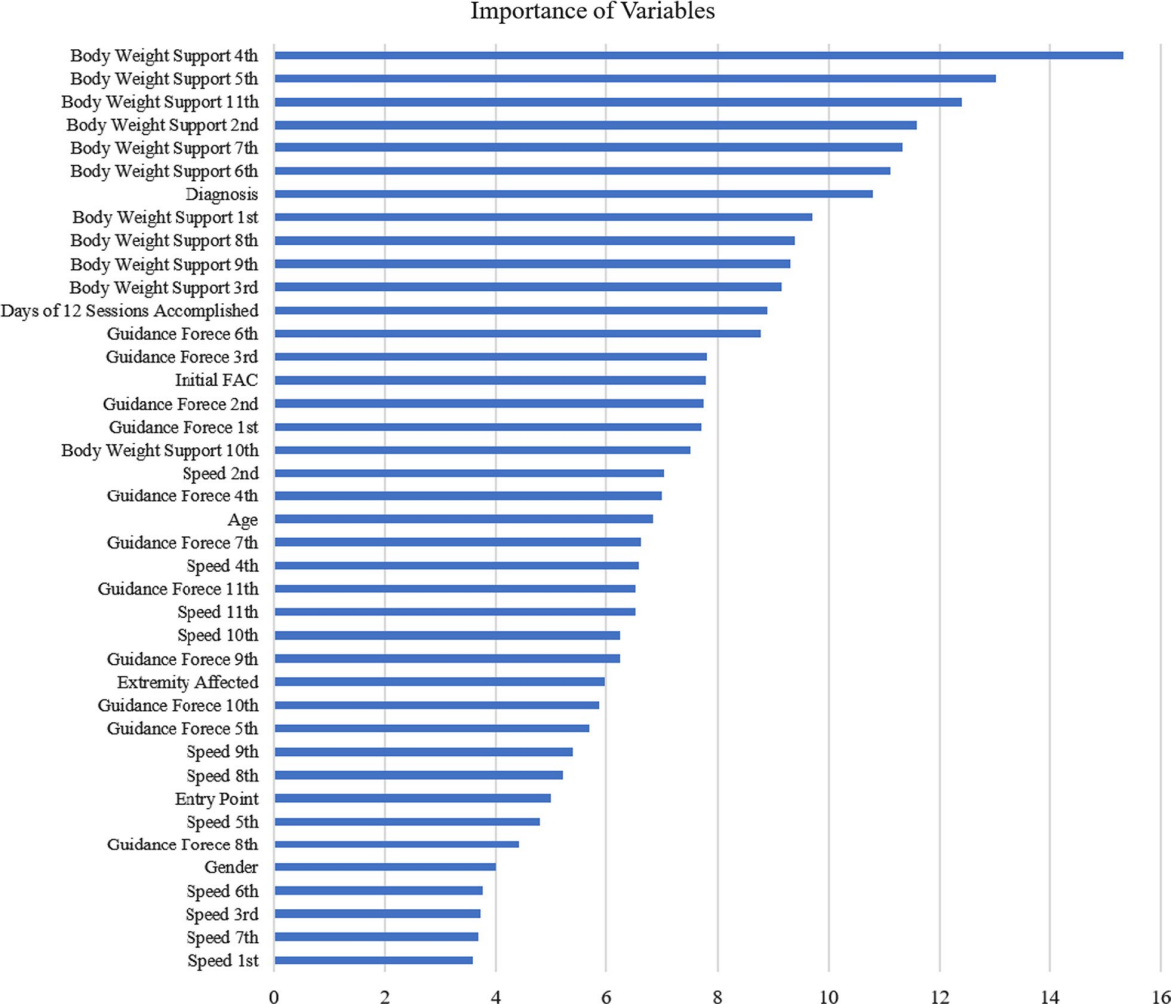
Variable ranking based on the discriminative power to predict the effectiveness of robot-assisted gait training (RAGT) for functional gait recovery

Since incorporating all sessions in the RF algorithm performed the best according to the above experimental results, further investigations of variable importance ranking and clinical interpretations in this section were in accordance with results of the RF using all sessions. Figure 2 demonstrates the importance of the ranking of variables according to their predictive power to distinguish the improvement and no-improvement groups using RAGT. From the ranking according to the MDA, BW support played a more-important role than the other variables of speed and GF with Lokomat. BW support levels of the fourth and fifth sessions were the most important variables to predict improvements in the FAC. For clinical variables, the diagnosis and days required to complete 12 sessions were two important variables for prediction.

**Fig. 3:**
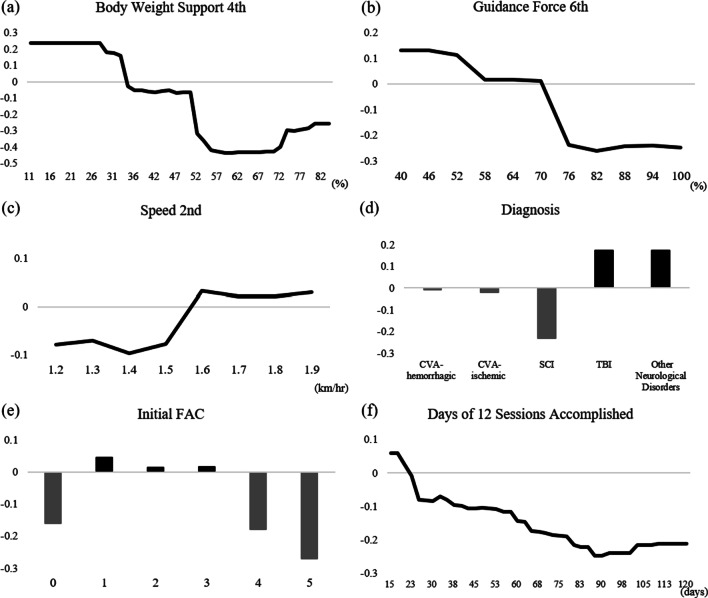
Partial dependence plots of important variables

In addition to the ranking of all input variables, a partial effect analysis using the RF algorithm was also used to individually evaluate the influence of each variable on outcomes. Figure 3 illustrates partial effect plots that depict the effects of selected variables on improving functional gait recovery. Positive values on the y-axis indicate that the values of the independent variables are more likely to be positive, while negative values are less likely to be positive. Obviously, zero indicates the absence of an average influence on the class probability.

In Fig. 3(a), BW support variables showed negative non-linear associations with outcomes. This suggests that if BW support of the 4th session was < 34%, i.e., reduced to nearly 1/3, the patient was more likely to show improvement in the FAC. In addition, as shown in Fig. 3(b), when the GF of the 6th session was < 70%, the patient was more likely to show improvement in the FAC, which indicated that the patient was more likely to see improvements. Moreover, as shown in Fig. 3(c), if the speed of RAGT was > 1.6 km/h, there was a higher propensity for progress in gait recovery. With regard to the diagnostic variables in Fig. 3(d), our results showed that patients with a traumatic brain injury (TBI) or other neurological disorders showed a higher propensity for improvements in FACs compared to those with a cardiovascular accident (CVA)-hemorrhagic, CVA-ischemic, or spinal cord injury (SCI). The initial FAC was another important variable. In Fig. 3e, patients with lower levels (i.e., 1–3) of FACs had a better propensity for functional gait recovery than those with higher levels (i.e., 4 or 5). Finally, when analyzing the time to complete 12 sessions (in days), spending fewer days to complete 12 sessions showed a higher propensity for improvement of functional gait recovery in Fig. 3(f). In summary, the experimental results demonstrated that our propensity analysis of RAGT parameters not only provides a medical interpretation for treatment but also corresponded well with clinical insights.

## Discussion

### Clinical relevance and analysis of the prediction model

Most motor rehabilitation trials with acute and subacute stroke patients reported equal improvements in the experimental and control groups, suggesting that newly onset stroke patients may spontaneously recover without a specific neurological rehabilitation approach [25]. A longitudinal study revealed that the level of functional and motor performance at 5 years post-stroke was equivalent to the level measured at 2 months [26]. In addition, that study indicated that a higher age and greater stroke severity negatively affected functional and motor recovery. However, only 29% patients in that study had regular physical therapy at 5 years post-stroke [26]. One can question whether current practice provides sufficient rehabilitation to judge what is possible for chronic stroke patients. The Lokomat system is capable of providing delicate control to challenge patients from FACs 0 to 5. Not surprisingly, in an RAGT study of patients with different neurological diagnoses and disease durations from 5 to 9 weeks at the baseline, those with low FAC scores showed the largest improvement in walking ability. Patients involved in our study were in the subacute or chronic stage. Among the 25 patients who showed an increased FAC at the 6th session, 48% had begun Lokomat training 3 months after disease onset. In our clinical experience, these results suggest that entry point and age may be less-important variables when RAGT such as Lokomat is used for gait training.

### Biomedical interpretation of selected discriminative variables

In this study, we provide a predictive model for ambulatory functional outcomes based on patients’ characteristics from early-stage quantitative data of robotic-based therapy on reductions in walking impairment. Contrary to previous studies, our study showed no significant group differences regarding patients’ ages or total days that they received Lokomat training, suggesting a wider effective range of robotic-based therapy in neurorehabilitation. Lokomat training often begins with 100% guidance to enforce a physiological gait pattern [27]. This could explain why only the GF of the 1st session showed no significant group differences. Findings from our descriptive statistics of categorical variables demonstrated that the entry point and diagnosis showed significant differences in outcome measures, which is in line with previous findings that early rehabilitation improves outcomes [28]. Human beings walk in a bipedal pattern; therefore, losing active control of more than one lower limb would require more assistive devices, and there would be less potential to progress to independent locomotion. The affected extremity may have different effects on the outcome measures.

### Parameter 1: the effect of BW support on the FAC

No previous studies indicated which adjustable robotic parameters produced greater benefits for gait recovery, and our study revealed that BW support was the most important RAGT variable for outcome measures compared to the GF and training speed. This result is supported by a previous study which reported that early treatment with partial weight-supported treadmill training could facilitate lower-extremity motor function and balance, and significantly improve kinematic data, such as hip flexion and extension angles, on gait recovery in stroke patients in the subacute phase [29].

To the best of our knowledge, no previous studies demonstrated relationships between BW support of RAGT and functional outcomes on a temporal basis. Our results indicated that the FAC was more likely to have progressed by the end of the 12th session if BW support gradually decreased as RAGT continued, and an improvement in the FAC at the end was also more likely. This result is in line with previous reports that during Lokomat training, reducing BW support and GF increased gluteus muscle and anterior tibialis muscle activation, while increasing the training speed enhanced muscle activation in both legs [16, 17].

### Parameter 2: the effect of guidance force on FAC

One case report of a stroke survivor showed that the self-selected and fast walking speed, 6-min walk test, timed up and go test, and lower extremity Fugl-Meyer score changed minimally after full-guidance robotic training, but improved considerably after 4 weeks of guided robotic training [30]. However, no specific cutoff points for training settings have yet been established. In our study, when the guidance force of the 6th session was < 70%, the FAC was likely to progress. Clinically, Lokomat training often begins with 100% guidance to enforce a physiological gait pattern, and it usually takes four sessions for a patient to regain kinematic control and add more activity to their movements [27]. Our results also suggested that the guidance force of the 6th session needs to be reduced to 70% to have more opportunity to progress in FACs after 12 sessions of RAGT.

### Parameter 3: the effect of speed on FAC

One of the most widely accepted facts in neurorehabilitation is that a high number of repetitions and frequent training sessions are crucial to reach the optimal recovery potential [31–33]. A previous study demonstrated that a high walking velocity resulted in a more-physiological gait pattern, and that variations in walking speed increased a patient’s attention and concentration [34]; however, no outcome-related analysis was done in that experiment. In our study, a novel and innovative relationship between speed and FAC progress was identified: a faster speed in the early sessions was correlated with a positive effect on the FAC. Our study also indicated that the best treatment frequency for FAC improvement was 12 sessions completed within 23 days.

### Clinical utility of the prediction model

Our results could support the concept that reductions in BW support and GF within the first few training sessions will increase the chance of seeing an improvement in the FAC. Due to the limited size of our input data, the present study demonstrates a general methodology of building a predictive model based on clinical and Lokomat data, but no specific guidelines can yet be established. The result can be seen as a proof-of-concept with an outlook as to what and how we would be able to use the algorithm in the future if we collected more data to separate patients into groups. The classifier is not to be used to stop a patient from RAGT training, but to inform and aid in planning further effective rehabilitation efforts with other strategies. At present, RAGT therapists can adjust the BW support, GF, and speed according to a patient’s progress while considering our prediction models (Figs. 2 and 3) at the same time. When patients gradually regain strength and control of their lower extremities, therapists can reduce the amount of weight supported and GF to promote more muscle activities as soon as is possible. When patients regain more temporal muscle activation, the GF can be further reduced to promote patients’ active participation in predefined gait patterns. Subsequently, when the performance of a patient is well adjusted to the above two parameters, the therapist can increase the training walking speed to increase repetitions and challenges.

## Study limitations

The current study has several limitations. First, we only analyzed whether three parameters of RAGT settings had positive effects on FAC outcomes. The Lokomat system is categorized as an exoskeletal-type stationary gait training system, and also provides delicate control of the range of motion and symmetrical or asymmetrical positions of the bilateral hips and knees depending on the patient’s condition and user management [35]. These settings also impact a patient’s progress. Developing models to predict more classes could make the estimates more impactful. However, due to the limited number of patients who could afford Lokomat treatment in this study, dividing the small dataset into additional classes to develop prediction models would have decreased the predictive performance. Second, all of the training parameters were collected at a median time point despite the fact that these settings are often dynamic and vary throughout the training sessions in clinical practice to challenge patients’ limits. Therefore, our results might not fully indicate the actual clinical situations. Third, in this study, the diagnoses and the onset of neurological diseases of the included patients ranged widely. Although our results indicated that a patient’s diagnosis was not among the top 10 most important variables, neurological patients substantially differed; therefore, each patient likely requires a personalized approach, which also includes individualized goals. In connection with this, the combination of RAGT parameters may be too diverse to obtain a clear picture of the prediction model for functional outcomes. We cannot elucidate the reason why some of our patients did not improve their walking capacity despite engaging in RAGT. The development of predictive biomarkers may help select patients who most likely will respond to a specific motor rehabilitation protocol in the future [36]. Fourth, conventional treatment plans and other interventions (i.e., repetitive transcranial magnetic stimulation [rTMS], injections for spasticity modulation) were not analyzed. Additional treatment approaches may affect patient outcomes to some extent. Another limitation of this study is that we only used FACs as a clinical outcome. Other measures such as the 6-min walking test (6MWT) or Berg balance scale may reflect relevant improvements achieved by RAGT. However, one study showed the concurrent validity of changes in FAC scores with changes in the 6MWT and was a good indicator of progress in walking function [36]. Finally, the no-improvement group was skewed toward the 0 FAC owing to fewer samples and higher fees for patients. In Taiwan, implementation of 12 sessions costs around US$2600, and this resulted in a lack of samples because some patients could not afford the fee. In the future, we will collect more patient data for analysis.

The prediction model with all sessions based on the random forest had the best performance, which may have been due to the relatively small sample size of our data. However, the results also showed that some of prediction models with fewer sessions had similar performances (shown in Additional file 1: Table S1). It was promising that the prediction model with fewer sessions probably had a potentially better performance, which will be confirmed in the future when more data are available.

## Conclusions and recommendations for future work

RAGT is a customized approach for patients with different conditions to regain walking ability. We developed a predictive model for ambulatory outcomes based on patient characteristics and quantitative data from early-stage robotic neurorehabilitation. In summary, this study offers possible adjustments of RAGT settings for Lokomat users to help patients with neurological disorders achieve their full potential in a relatively short period. Our results indicated that reducing body weight support and the guidance force within the first few training sessions increased the chance for improvements in the FAC. To obtain a more-precise and clearer predictive model, collecting more RAGT training parameters and analyzing them for each individual disorder is a possible approach to help clinicians achieve a better understanding of the most efficient RAGT parameters for different patients.

## Supplementary Information

**Additional file 1: Table S1.** Prediction performance of different machine learning algorithms with 10-fold cross validation using different numbers of input sessions to predict the improvement in FACs of the 12th session. **Table S2.** Comparison of predictive performance of mean AUC between the five machine learning algorithms by (a) ANOVA and (b) Tukey HSD test. **Fig. S1.** Prediction performance evaluated by means of AUC using clinical data and parameters from RAGT sessions into different machine learning algorithms to predict improvement or not in FAC by ten-fold cross-validation. **Table S3.** Comparison of predictive performance of mean accuracy between the five machine learning algorithms by (a) ANOVA and (b) Tukey HSD test. **Fig. S2.** Prediction performance evaluated by means of accuracy using clinical data and parameters from RAGT sessions into different machine learning algorithms to predict improvement or not in FAC by ten-fold cross-validation. **Table S4.** Comparison of predictive performance of mean sensitivity between the five machine learning algorithms by (a) ANOVA and (b) Tukey HSD test . **Fig. S3.** Prediction performance evaluated by means of sensitivity using clinical data and parameters from RAGT sessions into different machine learning algorithms to predict improvement or not in FAC by ten-fold cross-validation. **Table S5.** Comparison of predictive performance of mean specificity between the five machine learning algorithms by (a) ANOVA and (b) Tukey HSD test. **Fig. S4.** Prediction performance evaluated by means of specificity using clinical data and parameters from RAGT sessions into different machine learning algorithms to predict improvement or not in FAC by ten-fold cross-validation.

## Data Availability

The datasets generated and/or analyzed during the current study are not publicly available due to data not being de-identified and insufficiently organized. The data are available from the corresponding author on reasonable request.

## Abbreviations

ANOVA: Aanalysis of variance
AUC: Area under the receiver operating characteristic curve
BW: Body weight
CGT: Conventional gait training
FAC: Functional Ambulation Category
GF: Guidance force
HSD: Honest significant difference
MDA: Mean decrease in accuracy
RAGT: Robotic-assisted gait training
RF: Random forest
rTMS: Repetitive transcranial magnetic stimulation
SVM: Support vector machine
XGBoost: Extreme gradient boosting
