# Supplementary material for: Prediction of robotic neurorehabilitation functional ambulatory outcome in patients with neurological disorders
Source: J Neuroeng Rehabil. 2021 Dec 18;18:174. doi: 10.1186/s12984-021-00965-6 (PMC8684617; doi:10.1186/s12984-021-00965-6)
Supplement: Supplementary file 1 — Additional file 1: Table S1. Prediction performance of different machine learning algorithms with 10-fold cross validation using different numbers of input sessions to predict the improvement in FACs of the 12th session. Table S2. Comparison of predictive performance of mean AUC between the five machine learning algorithms by (a) ANOVA and (b) Tukey HSD test. Fig. S1. Prediction performance evaluated by means of AUC using clinical data and parameters from RAGT sessions into different machine learning algorithms to predict improvement or not in FAC by ten-fold cross-validation. Table S3. Comparison of predictive performance of mean accuracy between the five machine learning algorithms by (a) ANOVA and (b) Tukey HSD test. Fig. S2. Prediction performance evaluated by means of accuracy using clinical data and parameters from RAGT sessions into different machine learning algorithms to predict improvement or not in FAC by ten-fold cross-validation. Table S4. Comparison of predictive performance of mean sensitivity between the five machine learning algorithms by (a) ANOVA and (b) Tukey HSD test . Fig. S3. Prediction performance evaluated by means of sensitivity using clinical data and parameters from RAGT sessions into different machine learning algorithms to predict improvement or not in FAC by ten-fold cross-validation. Table S5. Comparison of predictive performance of mean specificity between the five machine learning algorithms by (a) ANOVA and (b) Tukey HSD test. Fig. S4. Prediction performance evaluated by means of specificity using clinical data and parameters from RAGT sessions into different machine learning algorithms to predict improvement or not in FAC by ten-fold cross-validation. [file 12984_2021_965_MOESM1_ESM.docx]

**Table S1. Prediction performance of different machine learning algorithms with 10-fold cross validation using different numbers of input sessions to predict the improvement in FACs of the 12^th^ session.**

| Algorithms | *i* | | AUC | | Accuracy | | Sensitivity | | Specificity | |
| --- | --- | --- | --- | --- | --- | --- | --- | --- | --- | --- |
|  |  |  | Mean | SD | Mean | SD | Mean | SD | Mean | SD |
| Random Forest | 1 | | 0.9496 | 0.0759 | 0.8497 | 0.0829 | 0.9667 | 0.1054 | 0.6833 | 0.1459 |
|  | 2 | | 0.9476 | 0.0539 | 0.8583 | 0.0710 | 0.9667 | 0.1054 | 0.7667 | 0.1165 |
|  | 3 | | 0.9520 | 0.0567 | 0.8644 | 0.0771 | 0.9667 | 0.1054 | 0.7333 | 0.1165 |
|  | 4 | | 0.9520 | 0.0646 | 0.8649 | 0.0903 | 1.0000 | 0.0000 | 0.8167 | 0.1459 |
|  | 5 | | 0.9591 | 0.0657 | 0.8772 | 0.0830 | 1.0000 | 0.0000 | 0.8167 | 0.1459 |
|  | 6 | | 0.9637 | 0.0599 | 0.8897 | 0.0869 | 1.0000 | 0.0000 | 0.8167 | 0.1459 |
|  | 7 | | 0.9702 | 0.0607 | 0.8898 | 0.0839 | 1.0000 | 0.0000 | 0.7833 | 0.1766 |
|  | 8 | | 0.9746 | 0.0522 | 0.8849 | 0.0827 | 1.0000 | 0.0000 | 0.8167 | 0.1459 |
|  | 9 | | 0.9720 | 0.0562 | 0.8842 | 0.0798 | 1.0000 | 0.0000 | 0.7833 | 0.1581 |
|  | 10 | | 0.9758 | 0.0527 | 0.8842 | 0.0773 | 1.0000 | 0.0000 | 0.8167 | 0.1459 |
|  | 11 | | 0.9813 | 0.0434 | 0.8789 | 0.0786 | 1.0000 | 0.0000 | 0.7667 | 0.1610 |
| Logistic Regression | 1 | | 0.7897 | 0.0998 | 0.7462 | 0.0983 | 0.7429 | 0.1526 | 0.7500 | 0.1179 |
|  | 2 | | 0.8036 | 0.1523 | 0.7609 | 0.1020 | 0.7714 | 0.1434 | 0.7500 | 0.1416 |
|  | 3 | | 0.7929 | 0.1462 | 0.7212 | 0.0976 | 0.7429 | 0.1526 | 0.7000 | 0.1532 |
|  | 4 | | 0.8548 | 0.1027 | 0.8365 | 0.0657 | 0.8714 | 0.1042 | 0.8000 | 0.1315 |
|  | 5 | | 0.8387 | 0.1534 | 0.8436 | 0.1373 | 0.9667 | 0.1054 | 0.7167 | 0.2229 |
|  | 6 | | 0.8371 | 0.1241 | 0.8776 | 0.1186 | 0.9667 | 0.1054 | 0.7833 | 0.1581 |
|  | 7 | | 0.7889 | 0.1088 | 0.7788 | 0.1406 | 0.9333 | 0.1405 | 0.6167 | 0.1766 |
|  | 8 | | 0.8649 | 0.1359 | 0.8436 | 0.1444 | 0.9333 | 0.1405 | 0.7500 | 0.1620 |
|  | 9 | | 0.8460 | 0.1061 | 0.8359 | 0.1112 | 0.9333 | 0.1405 | 0.7333 | 0.1405 |
|  | 10 | | 0.8796 | 0.0997 | 0.8359 | 0.0867 | 0.9333 | 0.1405 | 0.7333 | 0.1165 |
|  | 11 | | 0.9177 | 0.0606 | 0.8526 | 0.1010 | 0.9333 | 0.1405 | 0.7667 | 0.1405 |
| Support Vector Machine | 1 | | 0.8369 | 0.0877 | 0.7870 | 0.1113 | 0.8071 | 0.1499 | 0.7833 | 0.1372 |
|  | 2 | | 0.8683 | 0.1088 | 0.7923 | 0.1096 | 0.8048 | 0.1734 | 0.8167 | 0.0946 |
|  | 3 | | 0.8710 | 0.1105 | 0.8060 | 0.1105 | 0.8690 | 0.1889 | 0.8167 | 0.0946 |
|  | 4 | | 0.9000 | 0.1094 | 0.8141 | 0.1183 | 0.8690 | 0.1889 | 0.8167 | 0.0946 |
|  | 5 | | 0.9075 | 0.1113 | 0.8194 | 0.1167 | 0.8524 | 0.1833 | 0.8500 | 0.0946 |
|  | 6 | | 0.9313 | 0.1110 | 0.8387 | 0.1152 | 0.8690 | 0.1528 | 0.8667 | 0.1054 |
|  | 7 | | 0.9262 | 0.1252 | 0.8359 | 0.1193 | 0.8690 | 0.1528 | 0.8833 | 0.1125 |
|  | 8 | | 0.9234 | 0.1237 | 0.8442 | 0.1258 | 0.8690 | 0.1528 | 0.9000 | 0.1165 |
|  | 9 | | 0.9234 | 0.1166 | 0.8331 | 0.1165 | 0.8690 | 0.1528 | 0.8667 | 0.1315 |
|  | 10 | | 0.9210 | 0.1204 | 0.8331 | 0.1245 | 0.8857 | 0.1365 | 0.8333 | 0.1361 |
|  | 11 | | 0.9187 | 0.1204 | 0.8359 | 0.1239 | 0.8548 | 0.1655 | 0.8500 | 0.1230 |
| Algorithms | | *i* | AUC | | Accuracy | | Sensitivity | | Specificity | |
|  |  |  | Mean | SD | Mean | SD | Mean | SD | Mean | SD |
| Decision Tree | | 1 | 0.7325 | 0.1509 | 0.6173 | 0.0678 | 0.7238 | 0.2237 | 0.6333 | 0.1721 |
|  |  | 2 | 0.7647 | 0.1557 | 0.6417 | 0.0512 | 0.7286 | 0.2005 | 0.6500 | 0.1459 |
|  |  | 3 | 0.7601 | 0.1181 | 0.6361 | 0.0604 | 0.7095 | 0.1040 | 0.6000 | 0.1610 |
|  |  | 4 | 0.7456 | 0.1356 | 0.6434 | 0.1052 | 0.7524 | 0.2127 | 0.6333 | 0.2582 |
|  |  | 5 | 0.7202 | 0.1507 | 0.6280 | 0.1067 | 0.6881 | 0.1997 | 0.6500 | 0.2415 |
|  |  | 6 | 0.7038 | 0.1497 | 0.6308 | 0.0919 | 0.7071 | 0.1338 | 0.6667 | 0.2079 |
|  |  | 7 | 0.7002 | 0.1278 | 0.6203 | 0.0801 | 0.6762 | 0.1129 | 0.6333 | 0.2049 |
|  |  | 8 | 0.7058 | 0.1388 | 0.6231 | 0.0844 | 0.6595 | 0.0985 | 0.6667 | 0.2222 |
|  |  | 9 | 0.6950 | 0.1600 | 0.6231 | 0.0844 | 0.6262 | 0.2082 | 0.7167 | 0.2838 |
|  |  | 10 | 0.7058 | 0.1388 | 0.6231 | 0.0844 | 0.6595 | 0.0985 | 0.6667 | 0.2222 |
|  |  | 11 | 0.6950 | 0.1600 | 0.6177 | 0.0875 | 0.6262 | 0.2082 | 0.7167 | 0.2838 |
| XGBoost | | 1 | 0.9139 | 0.1152 | 0.7794 | 0.0770 | 0.9333 | 0.1405 | 0.7167 | 0.1125 |
|  |  | 2 | 0.9075 | 0.1082 | 0.7847 | 0.0672 | 0.9333 | 0.1405 | 0.7667 | 0.1405 |
|  |  | 3 | 0.9087 | 0.0832 | 0.7821 | 0.0639 | 0.9333 | 0.1405 | 0.7000 | 0.1721 |
|  |  | 4 | 0.8988 | 0.0898 | 0.8163 | 0.0774 | 0.9333 | 0.1405 | 0.7500 | 0.2115 |
|  |  | 5 | 0.9175 | 0.1077 | 0.8400 | 0.0690 | 0.9333 | 0.1405 | 0.8333 | 0.1571 |
|  |  | 6 | 0.9317 | 0.0927 | 0.8526 | 0.0618 | 0.9667 | 0.1054 | 0.8500 | 0.1459 |
|  |  | 7 | 0.9202 | 0.0933 | 0.8462 | 0.0644 | 0.9333 | 0.1405 | 0.8000 | 0.1721 |
|  |  | 8 | 0.9234 | 0.0915 | 0.8460 | 0.0646 | 0.9333 | 0.1405 | 0.8167 | 0.1834 |
|  |  | 9 | 0.9202 | 0.0933 | 0.8428 | 0.0689 | 0.9333 | 0.1405 | 0.8500 | 0.1657 |
|  |  | 10 | 0.9270 | 0.1020 | 0.8467 | 0.0708 | 0.9167 | 0.1416 | 0.8000 | 0.2194 |
|  |  | 11 | 0.9373 | 0.0703 | 0.8541 | 0.0720 | 0.9333 | 0.1405 | 0.8167 | 0.1657 |

SD: Standard Deviation

To investigate whether there are significant differences in means of AUC, accuracy, sensitivity, and specificity between the classifiers, ANOVA was incorporated to examine the difference, and our statistical analysis showed that there are significant differences (i.e., *p*-value < 0.05) in means of them among the five classifiers. Therefore, we further applied Tukey HSD (honestly significant difference) test for multiple comparisons. Except for specificity, result shows that there are significantly differences in means of AUC, accuracy, and sensitivity between decision tree and the other four classifiers. Random forest, XGBoost, support vector machines, and logistic regression showed no significantly difference by Tukey HSD test. Detailed statistical results of ANOVA and Tukey HSD tests were illustrated in Table S2(a)-S5(a) and Table S2(b)-S5(b), respectively.

**Table S2. Comparison of predictive performance of mean AUC between the five machine learning algorithms by (a) ANOVA and (b) Tukey HSD test**

(a)ANOVA

|  | Df | Sum Sq | Mean Sq | F value | Pr(>F) |
| --- | --- | --- | --- | --- | --- |
| Method | 4 | 0.5018 | 0.1254 | 12.4 | 0.0000 *** |
| Residuals | 45 | 0.4552 | 0.0101 |  |  |

(b) Tukey HSD test: multiple comparisons of means 95% family-wise confidence level

|  | diff | lwr | upr | p adj |
| --- | --- | --- | --- | --- |
| Logistic Regression-Decision Tree | 0.2226 | 0.0948 | 0.3504 | 0.0001 *** |
| Random Forest-Decision Tree | 0.2863 | 0.1585 | 0.4141 | 0.0000 *** |
| SVM-Decision Tree | 0.2236 | 0.0958 | 0.3514 | 0.0001 *** |
| XGBOOST-Decision Tree | 0.2423 | 0.1145 | 0.3701 | 0.0000 *** |
| Random Forest-Logistic Regression | 0.0637 | -0.0641 | 0.1915 | 0.6208 |
| SVM-Logistic Regression | 0.0010 | -0.1268 | 0.1288 | 1.0000 |
| XGBOOST-Logistic Regression | 0.0196 | -0.1082 | 0.1475 | 0.9922 |
| SVM-Random Forest | -0.0627 | -0.1905 | 0.0651 | 0.6347 |
| XGBOOST-Random Forest | -0.0440 | -0.1719 | 0.0838 | 0.8632 |
| XGBOOST-SVM | 0.0187 | -0.1092 | 0.1465 | 0.9936 |


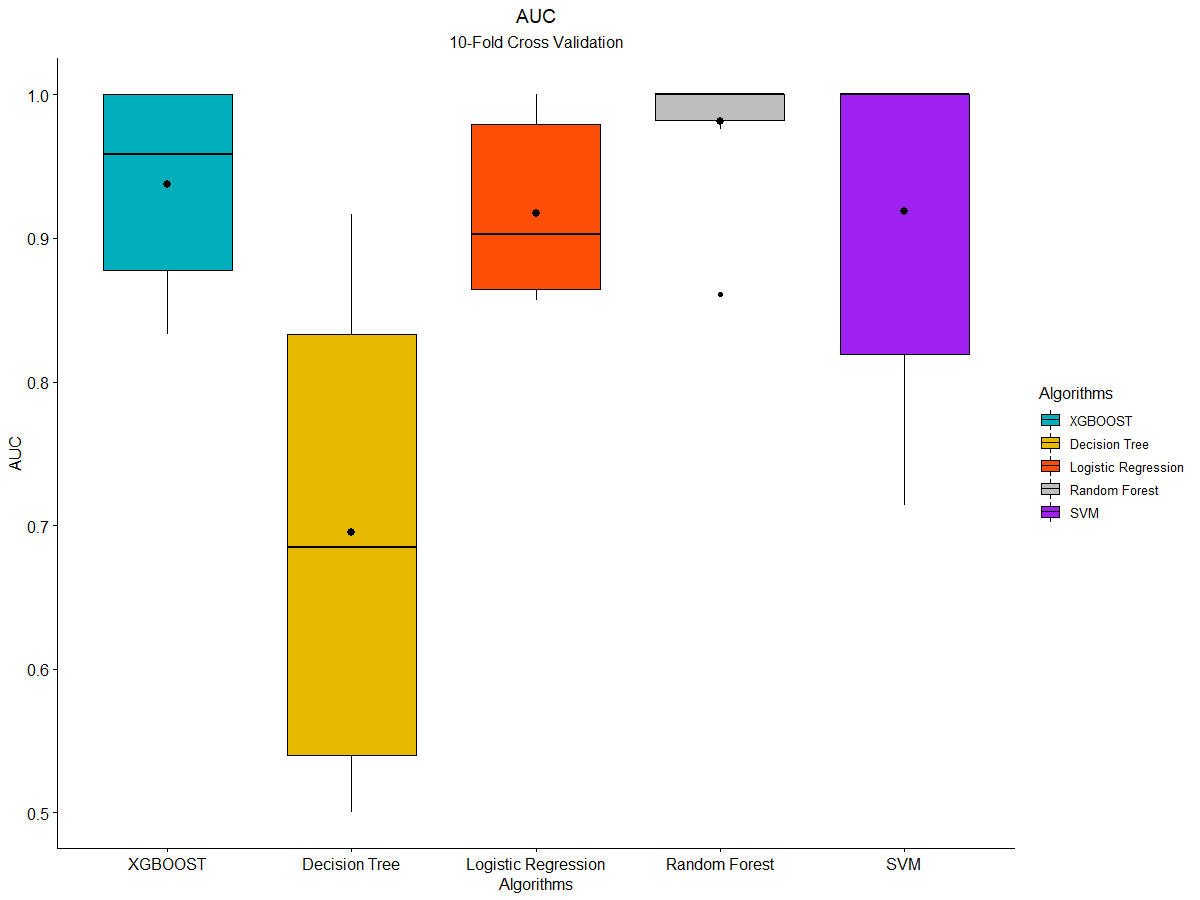


**Figure S1. Prediction performance evaluated by means of AUC using clinical data and parameters from RAGT sessions into different machine learning algorithms to predict improvement or not in FAC by ten-fold cross-validation**

**Table S3. Comparison of predictive performance of mean accuracy between the five machine learning algorithms by (a) ANOVA and (b) Tukey HSD test**

(a)ANOVA

|  | Df | Sum Sq | Mean Sq | F value | Pr(>F) |
| --- | --- | --- | --- | --- | --- |
| Method | 4 | 0.4612 | 0.1153 | 12.93 | 0.0000 *** |
| Residuals | 45 | 0.4012 | 0.0089 |  |  |

(b) Tukey HSD test: multiple comparisons of means 95% family-wise confidence level

|  | diff | lwr | upr | p adj |
| --- | --- | --- | --- | --- |
| Logistic Regression-Decision Tree | 0.2348 | 0.1148 | 0.3548 | 0.0000 *** |
| Random Forest-Decision Tree | 0.2612 | 0.1412 | 0.3812 | 0.0000 *** |
| SVM-Decision Tree | 0.2182 | 0.0982 | 0.3381 | 0.0001 *** |
| XGBOOST-Decision Tree | 0.2364 | 0.1164 | 0.3564 | 0.0000 *** |
| Random Forest-Logistic Regression | 0.0263 | -0.0936 | 0.1463 | 0.9705 |
| SVM-Logistic Regression | -0.0167 | -0.1366 | 0.1033 | 0.9947 |
| XGBOOST-Logistic Regression | 0.0016 | -0.1184 | 0.1216 | 1.0000 |
| SVM-Random Forest | -0.0430 | -0.1630 | 0.0770 | 0.8455 |
| XGBOOST-Random Forest | -0.0248 | -0.1447 | 0.0952 | 0.9764 |
| XGBOOST-SVM | 0.0182 | -0.1017 | 0.1382 | 0.9925 |


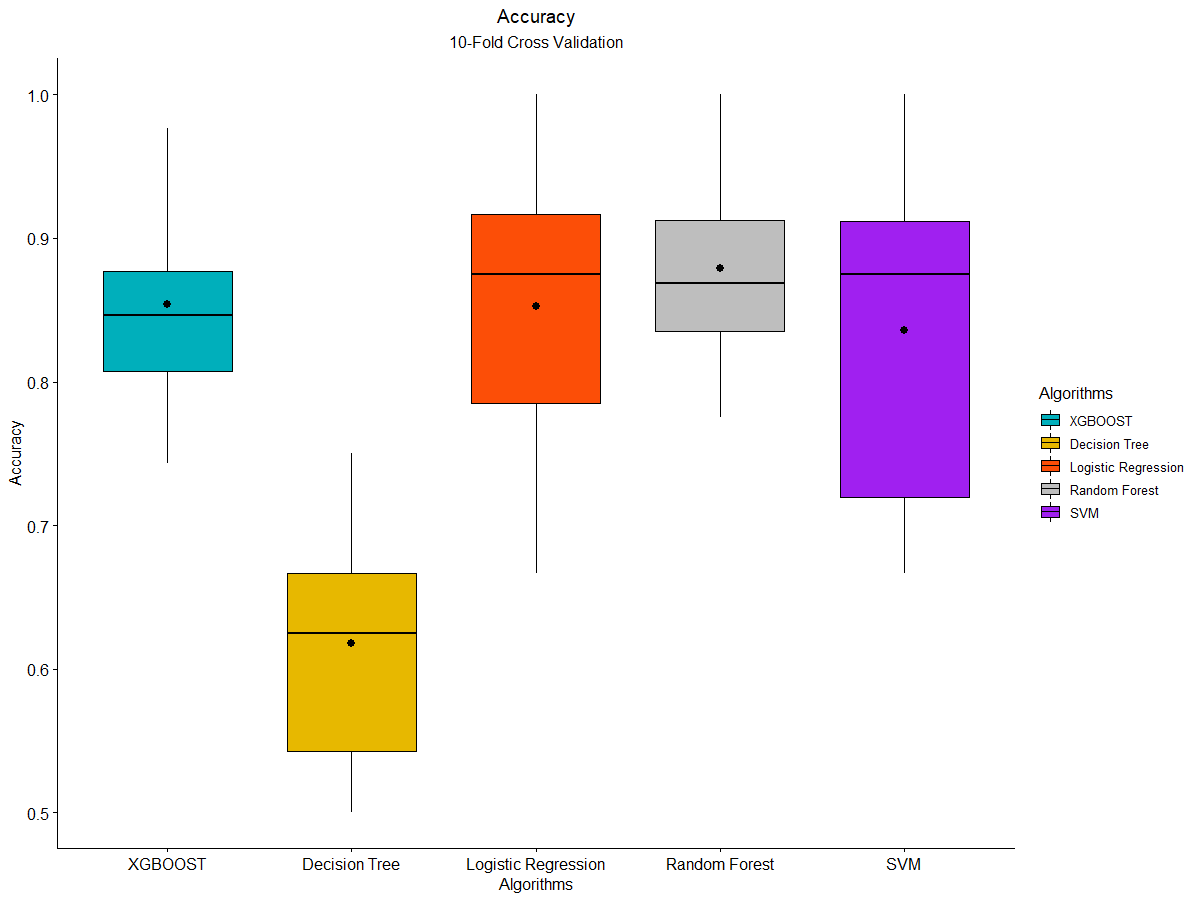


**Figure S2. Prediction performance evaluated by means of accuracy using clinical data and parameters from RAGT sessions into different machine learning algorithms to predict improvement or not in FAC by ten-fold cross-validation**

**Table S4. Comparison of predictive performance of mean sensitivity between the five machine learning algorithms by (a) ANOVA and (b) Tukey HSD test**

(a)ANOVA

|  | Df | Sum Sq | Mean Sq | F value | Pr(>F) |  |
| --- | --- | --- | --- | --- | --- | --- |
| Method | 4 | 0.4612 | 0.1153 | 12.93 | 0.0000 *** |  |
| Residuals | 45 | 0.4012 | 0.0089 |  |  |  |

(b) Tukey HSD test: multiple comparisons of means 95% family-wise confidence level

|  | diff | lwr | upr | p adj |
| --- | --- | --- | --- | --- |
| Logistic Regression-Decision Tree | 0.2348 | 0.1148 | 0.3548 | 0.0000 *** |
| Random Forest-Decision Tree | 0.2612 | 0.1412 | 0.3812 | 0.0000 *** |
| SVM-Decision Tree | 0.2182 | 0.0982 | 0.3381 | 0.0001 *** |
| XGBOOST-Decision Tree | 0.2364 | 0.1164 | 0.3564 | 0.0000 *** |
| Random Forest-Logistic Regression | 0.0263 | -0.0936 | 0.1463 | 0.9705 |
| SVM-Logistic Regression | -0.0167 | -0.1366 | 0.1033 | 0.9947 |
| XGBOOST-Logistic Regression | 0.0016 | -0.1184 | 0.1216 | 1.0000 |
| SVM-Random Forest | -0.0430 | -0.1630 | 0.0770 | 0.8455 |
| XGBOOST-Random Forest | -0.0248 | -0.1447 | 0.0952 | 0.9764 |
| XGBOOST-SVM | 0.0182 | -0.1017 | 0.1382 | 0.9925 |


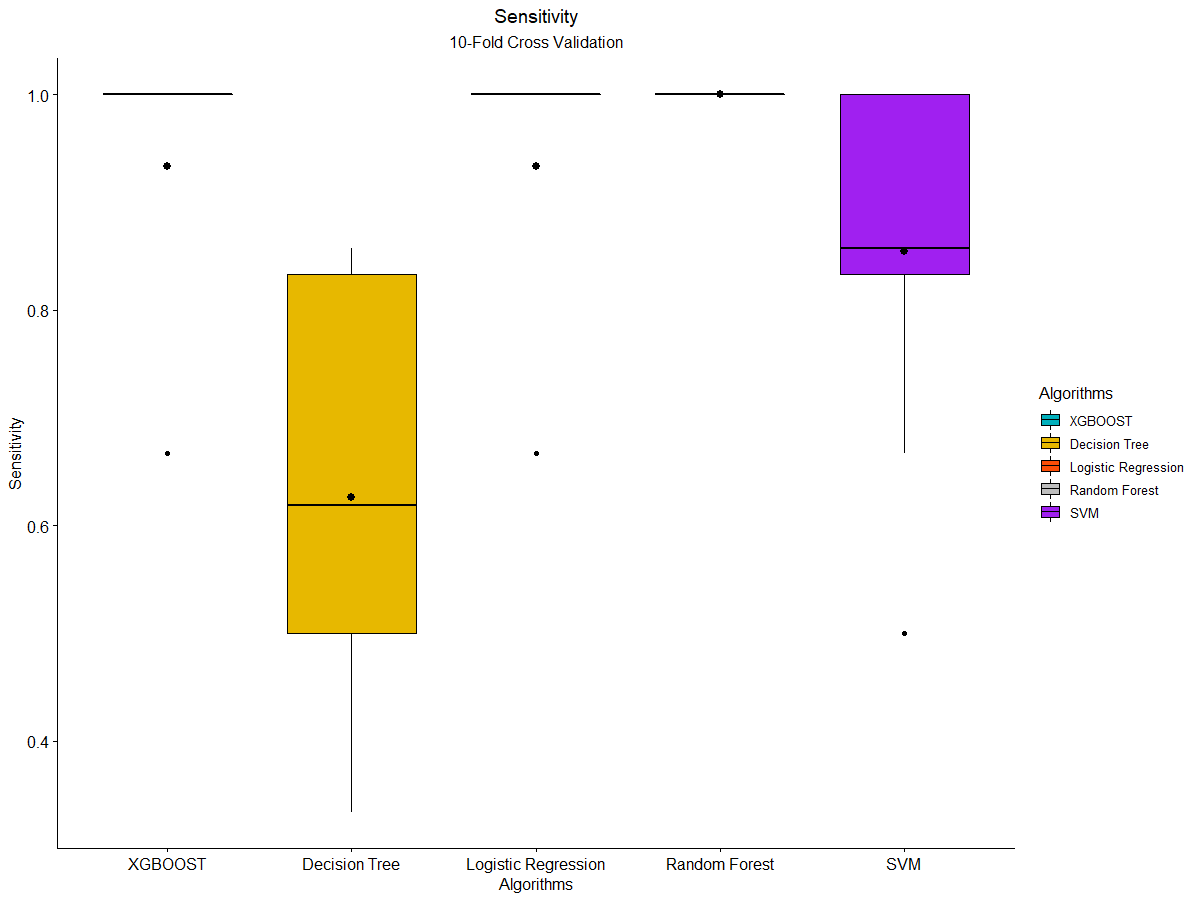


**Figure S3. Prediction performance evaluated by means of sensitivity using clinical data and parameters from RAGT sessions into different machine learning algorithms to predict improvement or not in FAC by ten-fold cross-validation**

**Table S5. Comparison of predictive performance of mean specificity between the five machine learning algorithms by (a) ANOVA and (b) Tukey HSD test**

(a)ANOVA

|  | Df | Sum Sq | Mean Sq | F value | Pr(>F) |
| --- | --- | --- | --- | --- | --- |
| Method | 4 | 0.1056 | 0.0264 | 0.782 | 0.5430 |
| Residuals | 45 | 1.5194 | 0.0338 |  |  |

(b) Tukey HSD test: multiple comparisons of means 95% family-wise confidence level

|  | diff | lwr | upr | p adj |
| --- | --- | --- | --- | --- |
| Logistic Regression-Decision Tree | 0.0500 | -0.1835 | 0.2835 | 0.9730 |
| Random Forest-Decision Tree | 0.0500 | -0.1835 | 0.2835 | 0.9730 |
| SVM-Decision Tree | 0.1333 | -0.1002 | 0.3668 | 0.4913 |
| XGBOOST-Decision Tree | 0.1000 | -0.1335 | 0.3335 | 0.7417 |
| Random Forest-Logistic Regression | 0.0000 | -0.2335 | 0.2335 | 1.0000 |
| SVM-Logistic Regression | 0.0833 | -0.1502 | 0.3168 | 0.8476 |
| XGBOOST-Logistic Regression | 0.0500 | -0.1835 | 0.2835 | 0.9730 |
| SVM-Random Forest | 0.0833 | -0.1502 | 0.3168 | 0.8476 |
| XGBOOST-Random Forest | 0.0500 | -0.1835 | 0.2835 | 0.9730 |
| XGBOOST-SVM | -0.0333 | -0.2668 | 0.2002 | 0.9941 |


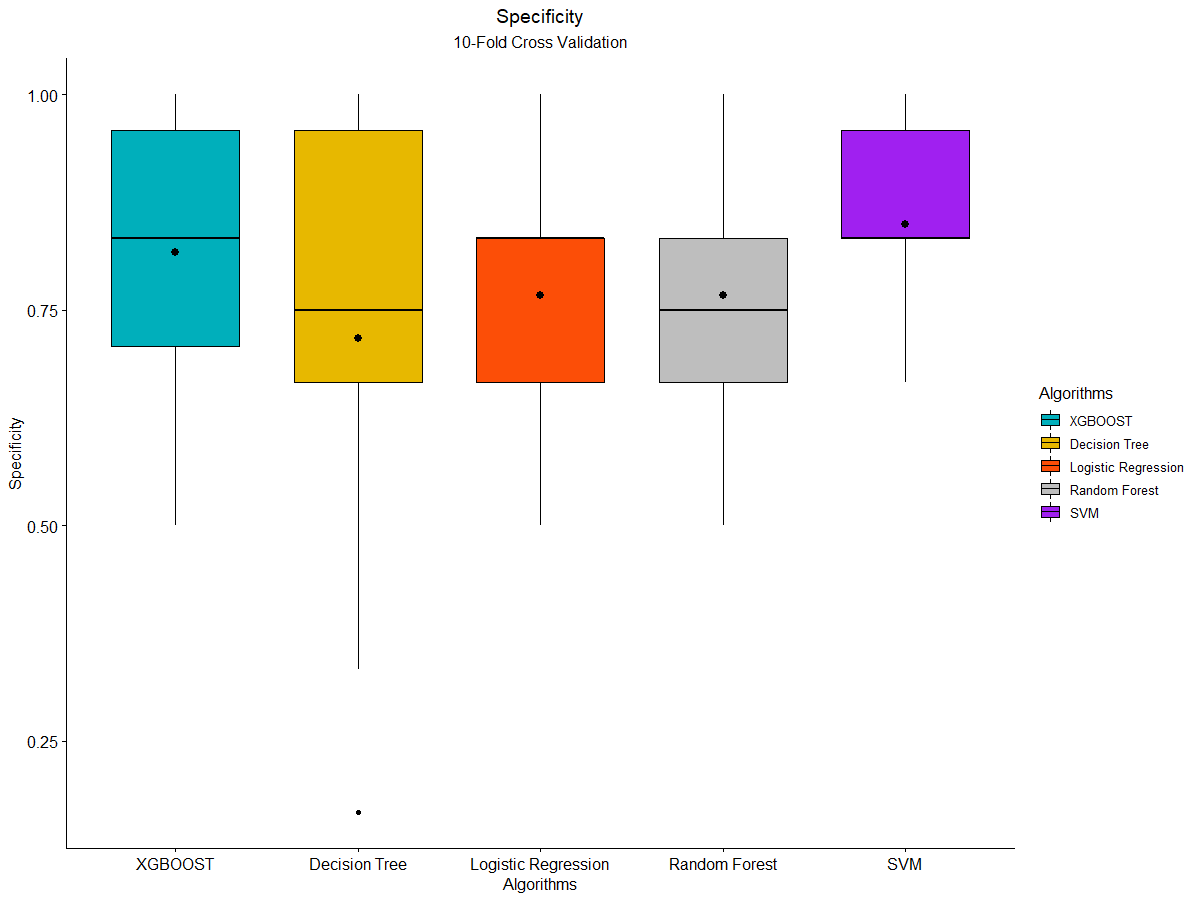


**Figure S4. Prediction performance evaluated by means of specificity using clinical data and parameters from RAGT sessions into different machine learning algorithms to predict improvement or not in FAC by ten-fold cross-validation**
